# A comprehensive analysis of adiponectin QTLs using SNP association, SNP cis-effects on peripheral blood gene expression and gene expression correlation identified novel metabolic syndrome (MetS) genes with potential role in carcinogenesis and systemic inflammation

**DOI:** 10.1186/1755-8794-6-14

**Published:** 2013-04-29

**Authors:** Yi Zhang, Jack W Kent, Michael Olivier, Omar Ali, Diana Cerjak, Ulrich Broeckel, Reham M Abdou, Thomas D Dyer, Anthony Comuzzie, Joanne E Curran, Melanie A Carless, David L Rainwater, Harald H H Göring, John Blangero, Ahmed H Kissebah

**Affiliations:** 1 TOPS Obesity and Metabolic Research Center, Department of Medicine, Medical College of Wisconsin, Milwaukee, Wisconsin, USA; 2 Human and Molecular Genetics Center, Medical College of Wisconsin, Milwaukee, Wisconsin, USA; 3 Department of Genetics, Texas Biomedical Research Institute, San Antonio, Texas, USA; 4 Department of Physiology, Medical College of Wisconsin, Milwaukee, Wisconsin, USA; 5 Department of Pediatrics, Medical College of Wisconsin, Milwaukee, Wisconsin, USA

**Keywords:** Adiponectin, Metabolic syndrome, Cancer risk, Inflammation

## Abstract

**Background:**

Metabolic syndrome (MetS) is an aberration associated with increased risk for cancer and inflammation. Adiponectin, an adipocyte-produced abundant protein hormone, has countering effect on the diabetogenic and atherogenic components of MetS. Plasma levels of adiponectin are negatively correlated with onset of cancer and cancer patient mortality. We previously performed microsatellite linkage analyses using adiponectin as a surrogate marker and revealed two QTLs on chr5 (5p14) and chr14 (14q13).

**Methods:**

Using individuals from 85 extended families that contributed to the linkage and who were measured for 42 clinical and biologic MetS phenotypes, we tested QTL-based SNP associations, peripheral white blood cell (PWBC) gene expression, and the effects of cis-acting SNPs on gene expression to discover genomic elements that could affect the pathophysiology and complications of MetS.

**Results:**

Adiponectin levels were found to be highly intercorrelated phenotypically with the majority of MetS traits. QTL-specific haplotype-tagging SNPs associated with MetS phenotypes were annotated to 14 genes whose function could influence MetS biology as well as oncogenesis or inflammation. These were mechanistically categorized into four groups: cell-cell adhesion and mobility, signal transduction, transcription and protein sorting. Four genes were highly prioritized: cadherin 18 (*CDH18*), myosin X (*MYO10*), anchor protein 6 of AMPK (*AKAP6*), and neuronal PAS domain protein 3 (*NPAS3*). PWBC expression was detectable only for the following genes with multi-organ or with multi-function properties: *NPAS3*, *MARCH6*, *MYO10* and *FBXL7*. Strong evidence of cis-effects on the expression of *MYO10* in PWBC was found with SNPs clustered near the gene’s transcription start site. *MYO10* expression in PWBC was marginally correlated with body composition (p= 0.065) and adipokine levels in the periphery (p = 0.064). Variants of genes *AKAP6*, *NPAS3*, *MARCH6* and *FBXL7* have been previously reported to be associated with insulin resistance, inflammatory markers or adiposity studies using genome-wide approaches whereas associations of *CDH18* and *MYO10* with MetS traits have not been reported before.

**Conclusions:**

Adiponectin QTLs-based SNP association and mRNA expression identified genes that could mediate the association between MetS and cancer or inflammation.

## Background

MetS is a cluster of phenotypes characterized by preferential deposition of fat in the abdominal/visceral region, insulin resistance, dyslipidemia, increased blood pressure and increased plasma levels of adipokines/cytokines [1]. Its complications include increased blood pressure/hypertension, glucose intolerance and type 2 diabetes. MetS has been associated with several forms of cancer including breast, endometrial, cervical, ovarian, esophageal, colon, rectal, pancreatic, hepatic, biliary and kidney cancers [2]. Overweight/obesity by itself accounts for 14% of cancer deaths in men and 20% in women [2]. Childhood cancer survivors are also known to present features of the MetS in later life [3].

Adiponectin is an adipocyte-produced kinin encoded by the gene apM1 (adipose most abundant transcript 1) at chr3q27 [4]. Adiponectin circulates in two forms, the low molecular weight (LMW) dimers, and the high molecular weight (HMW) oligomers. Tissue specificity of adiponectin *n*-mers is determined by their relative affinity for receptors (AdipoR1 and R2). AdipoR1 has a higher affinity for HMW. It is ubiquitously expressed but highly enriched in skeletal muscle. AdipoR2 can bind both forms and is mainly expressed in the liver [5]. Adiponectin has been shown to enhance insulin sensitivity and to exert anti-diabetogenic and anti-atherogenic functions as well as anti-inflammatory and anti-angiogenic activities [6-8]. Its plasma levels are inversely correlated with increased risk for obesity-related malignancies [9].

To explore possible genetic mechanisms linking MetS pathways with severe adverse outcomes such as development of cancer, we previously conducted a genome-wide linkage study using microsatellite markers at approximately 10 centiMorgan (cM) intervals in 1,100 individuals from 170 nuclear families (out of 85 extended families) of predominantly Northern European ancestry. This study identified two highly significant quantitative trait loci (QTLs) on chr5p14 and chr14q13 [10]. In the present study, we tested for genes harbored within these QTLs that could account for the association between MetS and carcinogenesis or systemic inflammation. For this purpose, we evaluated 1,137 individuals from the 85 extended families that contributed to the original linkage signals and who were phenotyped for 42 clinical and/or biological MetS traits (described in Results). We then performed QTL-focused SNP association and PWBC target gene expression analyses to identify those genes predisposing for this association.

## Methods

### Subjects and phenotypes

The present study cohort included 1,137 individuals from 85 extended families. Details of their recruitment and ascertainment traits procedures have been previously described [11,12]. Recruitment was initiated via an obese proband (BMI ≥ 30) with minimal availability of one obese and one never-obese (BMI ≤ 27) siblings and at least one, preferably both, parents. The clinical phenotypic components, which included weight, height, BMI, waist circumference (WC), hip circumference (HC), waist to hip ratio (WHR), fasting glucose (FG), fasting insulin (FI), insulin to glucose ratio (IGR), homeostasis model assessment (HOMA), plasma triglycerides (TG), total cholesterol (TC), LDL-cholesterol (LDL-c) and calculated LDL-cholesterol levels (cal. LDL-c), HDL-cholesterol (HDL-c), systolic and diastolic blood pressure (sBP and dBP) and pulse, as described [11]. The biological traits included total fat mass both in kilogram and percentage (Fatkg and Fatpct) and total fat free mass both in kilograms and as percentage of total body mass (Leankg and Leanpct) by DXA (Dual-emission X-ray absorptiometry) [13]; total abdominal, visceral and subcutaneous fat size (TAF, VF and SubQF) by computed tomography (CT) scans of the fourth lumbar spine [14]; respiratory quotient (RQ) and resting energy expenditure (REE) measured in resting subjects using a Deltatrac Indirect Calorimeter (Sensor Medics, VIASYS Healthcare, Conshohocken, PA) after a 10 hr fast; insulin/glucose responsiveness indices: insulin sensitivity (SI), glucose effectiveness (SG), acute insulin response to glucose (AIR_G_) and disposition index (DI) by Minimal Model [15]; lipids/lipoprotein sizing [HDL median diameter (HMED), LDL-cholesterol median diameter (LMEDn), LDL-cholesterol dominant peak diameter (LDLppd) and apoB-containing non-HDL median diameter (BMED) which includes VLDL, ILDL, LPα and LDL] measured by polyacrylamide gradient gel electrophoresis [16]; circulating levels of adiponectin, leptin by a double antibody, equilibrium RIA (Millipore Corporation, Billerica, MA); and TNF-alpha, interleukin-1beta (IL-1β) and interleukin-6 (IL-6)] measured as described [17]. All study procedures for adults and children were approved by the Institutional Review Boards of the Medical College of Wisconsin and Children’s Hospital of Wisconsin, respectively. Informed consent was obtained from the participating subjects or guardians of participating minors.

### SNP genotyping and data cleaning

Genomic DNA was extracted and prepared from whole blood using commercial kits (Puregene, Minneapolis, MN). Genome-wide SNP genotyping was performed using Affymetrix Genome-Wide Human SNP 6.0 arrays and SNP calls were generated by Genotype Console 3.2. Individuals with fewer than 95% of all available markers called were excluded. 869,222 autosomal SNPs were prepared by Preswalk and checked for Mendelian consistency with SimWalk2. A SNP was eliminated if: 1) fewer than 95% of the cohort were typed successfully; 2) the SNP was monoallelic; 3) the SNP had more than two alleles; 4) fewer than five copies of the SNP existed in the current study cohort. Hardy-Weinberg equilibrium (HWE) was tested for each SNP using SOLAR [18]; SNPs with excessive deviation from HWE (p < 10^-8^) were excluded. Missing SNP data were imputed with MERLIN [19].

### Transcriptional profiling

Genome-wide transcriptional profiles of a subset of the SNP genotyping cohort (369 individuals from 55 nuclear families) were obtained as previously described [20] with modifications. Briefly, for each individual 2.5 ml blood was collected into a PAXgene® Blood RNA Tube (BD, Franklin Lakes, NJ) following an overnight fast. Total RNA was isolated from each tube using the PAXgene Blood RNA Kit (Qiagen, Valencia, CA) and anti-sense RNA (aRNA) was synthesized using the MessageAmp II-Biotin aRNA kit (Ambion, Austin, TX). A total of 1.5 μg aRNA was hybridized to Illumina HumanWG-6 version 2 or version 3 chips (Illumina, San Diego, CA) and expression detected on the Illumina® BeadArray™ 500GX Reader. Illumina GenomeStudio software (version 2010.3) was used for preliminary data analysis with standard background normalization. The data discussed in this publication have been deposited in NCBI’s Gene Expression Omnibus [21] and are accessible through GEO Series accession number GSE45987 (http://www.ncbi.nlm.nih.gov/geo/query/acc.cgi?acc=GSE45987).

### Statistical analysis

#### Measured genotype analysis

In SOLAR, each SNP genotype was converted to a covariate measure equal to 0, 1 or 2 copies of the minor allele (or, for missing genotypes, a weighted covariate based on imputation). These covariates were included in variance-components mixed models for measured genotype analyses [22] versus null models that incorporated the random effect of kinship and fixed effects such as age, age^2^, sex and their interactions. Individual scores from a principal components analysis of representative SNPs were also included to correct for possible population stratification [23].

Within chromosomal regions showing the strongest prior evidence of linkage (as logarithm of odds, LOD, score) with MetS traits in our MRC-OB cohort, we selected all available SNPs on the Affymetrics 6.0 array that mapped within a 1-LOD confidence interval of the maximum LOD score in each region. Each SNP covariate was tested independently in a 1 degree of freedom likelihood ratio test. To take into account the linkage disequilibrium between SNPs in each region, we calculated the effective number of independent SNPs (N_eff_) using the method of Moskvina & Schmidt [24]. Critical p-values were calculated using Bonferroni/Šidák correction for each linkage region based on its N_eff_.

#### Gene expression analysis

Microarray data were available in two batches, one based on Version 2 arrays (48,701 probes, 307 samples) and the other on Version 3 (48,803 probes, 230 samples). To guard against possible batch effects and probe differences, each batch was analyzed separately: The number of probe transcripts detectable at p≤0.05 by BeadStudio software was counted, a false discovery rate (FDR) was computed across all probes, and transcripts detectable at 5% FDR were retained. Expression levels were log_2_ transformed and inverse-quartile normalized. Transformed and normalized expression levels for probes that mapped to the 1-LOD QTL regions were tested for association with phenotypes of interest in models that included the random effect of kinship. Gene-centric p-values were calculated by combining independent p-values from the two microarray batches and multiple probes using Stouffer’s weighted Z-score method [25] implemented in R.

## Results

### Intercorrelation between plasma adiponectin levels and MetS phenotypes

Based on their attributes in different biological pathways, we categorized our 42 measured MetS phenotypes into five groups: body composition, insulin responsiveness, lipids/lipoprotein profiles, cardiovascular performance, and adipokine and cytokines (Table 1). Means ± SD of the 42 MetS phenotypes and levels of their heritability have been previously described [26]. We found that plasma adioponectin levels were highly heritable (additive heritability, h^2^ = 0.48) in our study cohort. Intercorrelation analysis of leading MetS component phenotypes revealed significant phenotypic correlations to varied degrees (See the Additional file 1: Table S1). Plasma adiponectin is also significantly correlated with the majority of tested phenotypes (29 out of 41, nominal p<0.05). In particular, it is highly correlated with the MetS-defining [27,28] phenotypes WC (correlation p-value=1.86×10^-22^), FG (7.8×10^-4^), plasma TG (1.3×10^-8^), plasma HDL-c (5.2×10^-12^), sBP (1.55×10^-6^) and dBP (7.1×10^-3^). Overall, levels of adiponectin inversely correlate with a phenotypes representing adverse adipose distribution (weight, BMI, WC, HC, WHR, Fatkg, Fatpct and SubQF, VF and TAF), elevated fasting glucose levels and insulin resistance indices (FG, FI, IGR and HOMA), dyslipidemia (TG), cardiovascular malfunction (sBP, dBP and pulse) and leptin. On the contrary, adiponectin levels correlate positively with several phenotypes indexing leanness (Leanpct), insulin sensitivity (SI and SG) and HDL-c. These results indicate that adiponectin is a reliable and significant phenotypic surrogate of both the clinical and biological traits of MetS in our cohort.

**Table 1:** Correlations of plasma adiponectin with the other MetS phenotypes in our cohort

	**Phenotype**	**Correlation p-value**	**Inter-correlation (ρ)**
**Body composition**	Weight, kg	7.37×10^-17^	***−0.29***
	Height, cm	0.030	***−0.08***
	BMI, kg/m_2_	4.22×10^-16^	***−0.29***
	Waist circumference (WC), cm	1.90×10^-22^	***−0.34***
	Hip circumference (HC), cm	4.80×10^-16^	***−0.29***
	Waist to Hip ratio (WHR)	0.000025	***−0.15***
	Total Fat Mass (Fatkg), kg	0.00078	***−0.17***
	Total Fat Mass (Fatpct),%	0.032	***−0.11***
	Total Lean Mass (Leankg), kg	0.00013	***−0.20***
	Total Lean Mass (Leanpct), %	0.026	***0.11***
	Subcutaneous Fat (SubQF), g	0.0012	***−0.18***
	Visceral Fat (VF), g	2.04×10^-8^	***−0.30***
	Total Abdominal Fat (TAF), g	0.000039	***−0.23***
	Respiratory Quotient (RQ)	0.78	0.02
	Resting Energy Expenditure (REE), kcal/24 hrs	8.15×10^-7^	***−0.26***
	REE/weight, kcal/24hrs/kg	0.29	0.06
	REE/Lean mass (REE/LM), kcal/24hrs/kg	0.16	−0.08
**Insulin responsiveness**	Fasting Glucose (FG), mmol/l	0.00078	***−0.12***
	Fasting Insulin (FI),pmol/l	8.85×10-8	***−0.18***
	Insulin/glucose (IGR)	6.63×10^-6^	***−0.15***
	Homeostasis model assessment (HOMA)	3.40×10^-8^	***−0.19***
	Insulin Sensitivity (SI), (× 10^-4^/min/μU/ml)	1.28×10^-6^	***0.26***
	Glucose Effectiveness (SG), min^-1^	0.068	0.10
	Acute Insulin Response to glucose (AIR_G_), μU/ml × 10min	0.45	−0.04
	Disposition Index (DI), AUC (Insulin_0-10 min_) × SI	0.012	***0.14***
**Lipids/lipoprotein profiles**	Triglycerides (TG), mmol/l	1.30×10^-8^	***−0.20***
	Total Cholesterol (TC), mmol/l	0.50	−0.02
	LDL-cholesterol (LDL-c), mmol/l	0.42	−0.03
	Calculated LDL-C (cal. LDL-c), mmol/l	0.92	0.00
	HDL-cholesterol (HDL-c), mmol/l	5.17×10^-12^	***0.25***
	HMED, nm	0.000020	***0.18***
	LMEDn, nm	0.000851	***0.14***
	LDLppd, nm	4.43×10^-6^	***0.19***
	BMED, nm	0.058	0.08
**Cardiovascular performance**	Systolic Blood Pressure (sBP), mmHg	1.55×10^-6^	***−0.16***
	Diastolic Blood Pressure (dBP), mmHg	0.0071	***−0.09***
	Pulse, beats/min	0.023	***−0.08***
**Adipokine and cytokines**	Leptin, ng/ml	5.64×10^-11^	***−0.23***
	TNF-alpha, pg/ml	0.24	0.04
	Interleukin-1 beta (IL-1β), pg/ml	0.89	0.01
	Interleukin-6 (IL-6), pg/ml	0.81	−0.01

The pair-wise intercorrelation (ρ) and its respective significance (p-value) between plasma adiponectin levels and each of the other MetS phenotypes are shown. Inter-correlations that passed the nominal significance cutoff (p<0.05) are depicted in bold italic.

### SNP genotype/phenotype association

Consistent with common practice, we defined confidence regions for the previously defined adiponectin linkage peaks as the physical positions adjacent to the peak showing linkage evidence of at least LOD score - 1: 5p14 [peak LOD=4.1; chr5: 9,792,000-23,021,100bp (NCBI36/hg18)] and 14q13 [peak LOD=3.2; chr14: 23,131,000-36,761,868bp (NCBI36/hg18)]. Haplotype-tagging SNPs located within these regions were tested for statistical association with each of the 42 MetS phenotypes. To account for multiple testing within QTLs while accounting for linkage disequilibrium (LD) among SNPs, QTL-specific significance thresholds were calculated based upon Bonferroni/Šidák correction for the effective number of independent SNPs at 5p14 (n_SNPs_ =3540, n_effective_= 2429.61, p_α=0.05_= 2.11×10^-5^) and 14q13 (n_SNPs_=3794, n_effective_ =2760.37, p_α=0.05_=1.86×10^-5^). SNPs that passed region-specific significance thresholds for respective traits and/or the highest nominally significant SNP of each trait for each trait are shown in Tables 2 and 3.

**Table 2:** MetS SNP association with phenotypes and their gene annotation at 5p14 QTL

**Variant**	**Minor allele**	**MAF**	**-LOG(p)**	**Associated phenotypes**	**Nearest gene**	**Location**	**Annotation**
rs10061119	T	0.26	**5.07**	Weight	*CDH18*	intron	Cell Cell adhesion
rs13357704	A	0.26	**5.07**	Weight	*CDH18*	intron	Cell Cell adhesion
rs10805723	G	0.25	**5.03**	Weight	*CDH18*	intron	Cell Cell adhesion
rs16887308	T	0.26	**5.03**	Weight	*CDH18*	intron	Cell Cell adhesion
rs4386736	A	0.26	**5.03**	Weight	*CDH18*	intron	Cell Cell adhesion
rs6879762	C	0.26	**5.03**	Weight	*CDH18*	intron	Cell Cell adhesion
rs10062513	G	0.25	**4.68**	Weight	*CDH18*	intron	Cell Cell adhesion
rs13355121	C	0.25	**4.68**	Weight	*CDH18*	intron	Cell Cell adhesion
rs10062864	A	0.25	**4.57**	Weight	*CDH18*	intron	Cell Cell adhesion
rs163282	A	0.26	4.27	Height	*FAM134B*	intron	Neuron maintenance
rs10061119	T	0.26	**4.82**	BMI	*CDH18*	intron	Cell Cell adhesion
rs13357704	A	0.26	**4.82**	BMI	*CDH18*	intron	Cell Cell adhesion
rs10805723	G	0.25	**4.77**	BMI	*CDH18*	intron	Cell Cell adhesion
rs16887308	T	0.26	**4.77**	BMI	*CDH18*	intron	Cell Cell adhesion
rs4386736	A	0.26	**4.77**	BMI	*CDH18*	intron	Cell Cell adhesion
rs6879762	C	0.26	**4.77**	BMI	*CDH18*	intron	Cell Cell adhesion
rs13355121	C	0.25	**4.52**	BMI	*CDH18*	intron	Cell Cell adhesion
rs10062864	A	0.25	**4.43**	BMI	*CDH18*	intron	Cell Cell adhesion
rs10061119	T	0.26	**4.66**	WC	*CDH18*	intron	Cell Cell adhesion
rs13357704	A	0.26	**4.66**	WC	*CDH18*	intron	Cell Cell adhesion
rs10805723	G	0.25	**4.57**	WC	*CDH18*	intron	Cell Cell adhesion
rs16887308	T	0.26	**4.57**	WC	*CDH18*	intron	Cell Cell adhesion
rs4386736	A	0.26	**4.57**	WC	*CDH18*	intron	Cell Cell adhesion
rs6879762	C	0.26	**4.57**	WC	*CDH18*	intron	Cell Cell adhesion
rs995021	C	0.49	3.57	HC	*CDH18*	intron	Cell Cell adhesion
rs1864220	G	0.07	3.45	WHR	*FBXL7*	intron	Protein sorting
rs860545	T	0.16	2.89	Fatkg	*MARCH11*	intergenic	Protein sorting
rs10223312	T	0.01	3.52	Fatpct	*mRNA BC033144*	intergenic	unknown function
rs17707882	A	0.04	**4.47**	Leankg	*MYO10*	intron	Cell Cell adhesion
rs10223312	T	0.01	3.54	Leanpct	*mRNA BC033144*	intergenic	unknown function
rs1840870	C	0.44	2.44	SubQF	*EST BG220738*	intergenic	unknown function
rs11134371	T	0.33	4.04	VF	*LOC285692*	intron	unknown function
rs17275322	G	0.03	2.82	TAF	*LOC285692*	intron	unknown function
rs12652510	C	0.19	3.11	RQ	*CDH18*	intron	Cell Cell adhesion
rs10065719	G	0.14	2.74	REE	*AY330599*	intergenic	tumor antigen
rs6859862	C	0.41	3.61	REE/weight	*DNAH5*	intron	motor protein
rs1809880	G	0.06	3.16	REE/Lean	*ROPN1L*	intron	sperm protein
rs7721328	G	0.17	3.57	FG	*mRNA TAG1*	intron	tumor antigen
rs2582660	C	0.18	2.88	FI	*MARCH11*	intergenic	Protein sorting
rs16902967	A	0.03	3.77	IGR	*DNAH5*	intergenic	*motor protein*
rs4327597	A	0.14	3.25	HOMA	*TAG*	intron	tumor antigen
rs831657	G	0.14	3.23	SI	*MARCH11*	intergenic	protein sorting
rs1551936	T	0.15	3.44	SG	*FAM134B*	intron	Neuron maintenance
rs2929724	C	0.50	3.30	AIR	*LOC285696*	intron	unknown function
rs10805650	G	0.33	3.11	DI	*EST BM682321*	intergenic	unknown function
rs16887451	C	0.07	3.10	TG	*mRNA BC028204*	intron	unknown function
rs17839277	T	0.03	3.55	TC	*mRNA BC028204*	intergenic	unknown function
rs12659663	A	0.25	4.17	LDL-c	*mRNA BC028204*	intron	unknown function
rs12659663	A	0.25	3.87	cal. LDL-c	*mRNA BC028204*	intron	unknown function
rs6883134	G	0.06	3.25	HDL-c	*TRIO mRNA*	intron	protein interaction
rs10065719	G	0.14	**4.48**	HMED	*AY330599*	intergenic	tumor antigen
rs10079252	G	0.49	4.30	LMEDn	*mRNA U92022*	intergenic	transposon Hsmar2
rs10073730	C	0.01	3.77	LDLppd	*MYO10*	intron	Cell Cell adhesion
rs6894869	C	0.01	**5.13**	BMED	*CTNND2*	intron	Cell Cell adhesion
rs5745297	A	0.07	3.58	sBP	*DAP*	3’ UTR	cell death
rs6450583	G	0.21	3.27	dBP	*mRNA BC028204*	intergenic	*unknown function*
rs1971391	A	0.44	3.10	Pulse	*mRNA AK130861*	intergenic	*unknown function*
rs31509	G	0.28	3.33	Adiponectin	*MYO10*	intron	Cell Cell adhesion
rs404639	G	0.06	4.29	Leptin	*CDH12*	intron	Cell Cell adhesion
rs6893920	G	0.22	4.04	TNF-alpha	*mRNA U92022*	intergenic	*transposon Hsmar2*
rs7730897	T	0.10	3.47	IL-1β	*MARCH11*	intergenic	protein sorting
rs17707882	A	0.04	3.77	IL-6	*MYO10*	intron	Cell Cell adhesion

SNPs within 1 LOD reduction from the linkage peak were tested for associations against the 42 phenotypes. Annotated genes and their known function were derived from the NCBI build 36 human genome assembly. Annotations were determined at the position and within 250kb up- and downstream of the associated SNP. Data shown in column “-Log10(p)” are levels of the highest association. SNP associations that exceed statistical QTL-specific significance threshold are bolded.

**Table 3:** MetS SNP association with phenotypes and their gene annotation at 14q13 QTL

**Variant**	**Minor allele**	**MAF**	**-LOG(p)**	**Associated phenotypes**	**Nearest gene**	**Location**	**Annotation**
rs1955850	G	0.33	2.95	Weight	*NOVA1*	intergenic	post-transcription regulation
rs2332524	G	0.10	2.87	Height	*STXBP6*	intergenic	exocytosis
rs1955850	G	0.33	3.75	BMI	*NOVA1*	intergenic	post-transcription regulation
rs6574794	A	0.06	3.08	WC	*NOVA1*	intergenic	post-transcription regulation
rs1956993	C	0.09	2.92	HC	*AKAP6*	intergenic	signal transduction
rs17096124	G	0.03	3.81	WHR	*PRKD1*	intron	signal transduction
rs8007613	G	0.28	2.88	Fatkg	*NPAS3*	intron	Transcription regulation
rs41449149	T	0.07	3.45	Fatpct	*PRKD1*	intergenic	signal transduction
rs1111533	C	0.05	3.53	Leankg	*NPAS3*	intron	Transcription regulation
rs41449149	T	0.07	3.55	Leanpct	*PRKD1*	intergenic	signal transduction
rs10137682	G	0.37	3.35	SubQF	*NPAS3*	intron	Transcription regulation
rs12888531	C	0.06	3.59	VF	*SLC25A21*	intron	solute carrier
rs10137682	G	0.37	3.39	TAF	*NPAS3*	intron	Transcription regulation
rs11623278	G	0.46	4.16	RQ	*AKAP6*	intergenic	signal transduction
rs11161057	A	0.42	3.42	REE	*MIR548AI*	intergenic	mircoRNA
rs10137682	G	0.37	3.50	REE/weight	*NPAS3*	intron	Transcription regulation
rs17414154	A	0.05	3.43	REE/Lean	*AKAP6*	intron	signal transduction
rs17112354	A	0.08	2.95	FG	*BC148262/MIR4307/LOC100505967/NOVA1*	intergenic	cDNA clone/mircoRNA/non coding RNA/paraneoplastic disease antigens
rs2105274	G	0.39	3.40	FI	*NOVA1*	intergenic	paraneoplastic disease antigens
rs1626390	C	0.11	3.48	IGR	*EGLN3*	intergenic	Transcription regulation
rs2105274	G	0.39	3.47	HOMA	*NOVA1*	intergenic	paraneoplastic disease antigens
rs1951758	T	0.36	3.21	SI	*MIR4307 / LOC100505967 / NOVA1*	intergenic	mircoRNA/non-coding RNA/paraneoplastic disease antigens
rs17114954	A	0.04	3.32	SG	*MIR548AI*	downstream	mircoRNA
rs8012040	G	0.46	3.53	AIR	*BC148262 /LOC100505967/ NOVA1*	intergenic	cDNA clone / non coding RNA/paraneoplastic disease antigens
rs10483452	C	0.27	3.82	DI	*PPP2R3C*	intron	signal transduction
rs8006023	A	0.05	4.60	TG	*NPAS3*	intron	transcription regulation
rs8004607	C	0.10	4.44	TC	*NPAS3*	intron	transcription regulation
rs712300	C	0.26	4.09	LDL-c	*EGLN3*	intergenic	transcription regulation
rs8004607	C	0.10	3.71	cal. LDL-c	*NPAS3*	intron	transcription factor
rs10136818	T	0.02	3.63	HDL-c	*STXBP6*	intergenic	exocytosis
rs12886242	G	0.12	2.61	HMED	*NPAS3*	intron	Transcription regulation
rs17103757	G	0.05	3.84	LMEDn	*BRMS1L*	intergenic	transcription regulation
rs847501	C	0.35	3.29	LDLppd	*BRMS1L*	intergenic	transcription regulation
rs10134570	C	0.02	3.83	BMED	*NPAS3*	intron	Transcription regulation
rs1951039	A	0.45	4.26	sBP	*STXBP6/NOVA1*	intergenic	exocytosis/paraneoplastic disease antigens
rs10498306	C	0.13	4.17	dBP	*MIR548AI/PRKD1*	intergenic	microRNA/signal transduction
rs8007613	G	0.28	3.04	Pulse	*NPAS3*	intron	Transcription regulation
rs9322942	A	0.33	3.43	Adiponectin	*SRP54*	intron	signal recognition particle 54kDal
rs17427680	C	0.05	3.12	Leptin	*G2E3*	intergenic	protein sorting
rs1440983	A	0.04	3.64	TNF-alpha	*PRKD1*	intergenic	signal transduction
rs11849533	C	0.17	3.67	IL-1B	*NPAS3*	intron	Transcription regulation
rs17406989	T	0.09	2.87	IL-6	*NPAS3*	intron	Transcription regulation

SNPs within 1 LOD reduction from the linkage peak were tested for associations against the 42 phenotypes. Annotated genes and their known function were derived from the NCBI build 36 human genome assembly. Annotations were determined at the position and within 250kb up- and downstream of the associated SNP. Data shown in column “-Log10(p)” are levels of the highest association. SNP associations that exceed statistical QTL-specific significance threshold are bolded.

### QTL5p14

**Figure 1:**
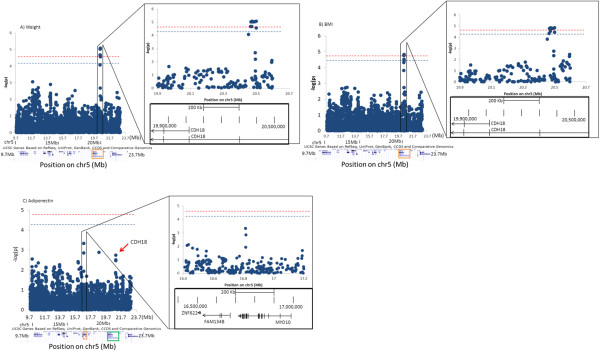
**Manhattan plots of SNP associations with MetS phenotypes within adiponectin QTL at 5p14.** Dark blue dots depict levels of association of identifier phenotypes with all SNPs in the region determined from 1 LOD reduction from the peak [chr5: 9,792,000-23,021,100 (NCBI36/hg18)]. Vertical axis represents minus logarithm of the p-values and horizontal represents the chromosomal position (Mb). Levels of QTL-wide significance thresholds are shown by the dash lines. Red lines indicate the significant level (p_α=0.05_= 2.11×10^-5^) and blue lines indicate suggestive level p_α=0.1_= 4.11×10^-5^). **A)** Association patterns of all the SNPs of the region with weight phenotype. Transcripts defined by UCSC genome browser (31, 32) are shown below the Manhattan plot, by blue bars. SNPs that show highest association with weight are located in gene CDH18 (framed in orange). **B)** SNP associations with BMI. **C)** SNP associations with plasma levels of adiponectin. SNPs with strongest associations are mapped to *MYO10*, shown framed in the lower panel and the blown-up picture. The second locus of peak signals (pointed by an arrow) is mapped to be 400kb upstream of CDH18, framed in green.

Fine-mapping of this region identified QTL-wide significant SNP associations with body composition phenotypes, including weight and BMI (Figure 1A and 1B). As shown in Table 2, 7 SNPs led by rs10061119 are mapped to an intronic region of the gene encoding cadherin 18 (*CDH18*). In addition, SNPs annotated near myosin X (*MYO10*), showed nominally significant associations with plasma adiponectin (Table 2 and Figure 1C). The second strongest SNP association cluster with adiponectin (Figure 1C, pointed by a red arrow) is intergenic and is approximately 400kb 5’ to the transcription start site of the cadherin-18 gene *CDH18* (Figure 1C, framed in green).

SNPs mapped to *CDH18* (cadherin 18) were also nominally associated with other MetS phenotypes including WC, HC and RQ. SNP rs404639 mapped to an intron of *CDH12*, another member of the cadherin family, and was associated with plasma leptin. SNP rs6894869 was significantly associated with lipid particle size phenotype BMED and mapped to an intron of *CTNND2*, which functions in the same pathway as cadherins, plays a crucial role in CNS development [29] and is a partner of the Alzheimer disease-associated gene presenilin-1 [30]. *MYO10* was also nominally associated with Leankg, LDLppd and IL-6. For the other MetS traits the strongest associations were with SNPs near several other genes with biological relevance to cancer. SNPs near *TAG*, a tumor antigen gene, were associated with HMED (high density lipid particle sizing), REE (resting energy expenditure), and insulin phenotypes FG and HOMA. SNPs mapped to *MARCH11* and *FBXL7*, two ubiquitin ligases involved in the pro-inflammation pathways, were associated with total fat mass, FI, SI and pro-inflammation marker IL-1beta (*MARCH11*), and with lean body mass (*FBXL7*).

### QTL14q13

**Figure 2:**
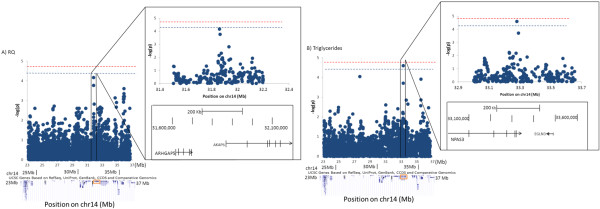
**Manhattan plots of SNP associations with MetS phenotypes within adiponectin QTL at 14q13.** Dark Blue dots depict levels of association of identifier phenotypes with all SNPs in the region determined from 1 LOD reduction from the peak [chr14: 23,131,000-36,761,868 (NCBI36/hg18)]. Vertical axis represents minus logarithm of the p-values and horizontal represents the chromosomal position (Mb). Levels of QTL-wide significance thresholds are shown by the dash lines. Red lines indicate the significant level (p_α=0.05_= p_α=0.05_=1.86×10^-5^) and blue lines indicate suggestive level p_α=0.1_= 3.72×10^-5^). **A)** Association patterns of all the SNPs of the region with RQ phenotype. Transcripts defined by UCSC genome browser (31, 32) are shown below the Manhattan plot, by blue bars. SNPs that show highest association with weight are located in gene AKAP6 (framed in red). **B)** SNP associations with triglycerides. SNPs that show highest associations with triglycerides are mapped to the transcription factor gene NPAS3.

This QTL region has a high density of genes with regulatory functions including signal transduction, transcription and post-transcription processing. Our fine-mapping identified SNPs associated with each of the 42 MetS phenotypes. Table 3 summarizes the SNPs most highly associated with each trait, the attributes of the variants, and the gene ontology of their annotated genes. Variants of *NPAS3*, a neuronal transcription factor thought to be involved in brain tumor suppression, were found to be associated with 11 of the MetS phenotypes (Fatkg, Leankg, SubQF, TAF, REE, TG, TC, LDL-c, pulse, IL1b and IL-6), suggesting a pleitropic effect (Table 3). Two of these associations (TG and TC) were borderline significant, marginally reaching the regional threshold (Table 3 and Figure 2A). SNPs near *BRMS1L*, a breast cancer suppressor gene, was associated with LMEDn and LDLppd. SNPs of *EGLN3*, a regulator of transcription factor HIF that affects apoptosis in hemangioblastoma and clear cell renal cancer, were associated with IGR and LDL-c. SNPs of *NOVA1*, a post-transcription processing of the GnRH paraneoplastic antigen, were associated at nominal significance with several phenotypes including weight, BMI, WC, FG, FI, HOMA, SI, AIR, and sBP.

Prominent SNP associations in this region were mapped to genes for signal transducers include kinases and kinase-related proteins *AKAP6* and *PRKD1* and the phosphotase PPP2R3C. SNPs of the gene encoding *AKAP6*, an anchor protein for AMPK, which may connect MetS with cancer, were associated with HC, RQ and REE/leanmass. SNPs of *PRKD1*, a kinase that phosphorylates and therefore activates E-cadherin functions, were associated with WHR, Fatpct, Leanpct, and TNF-alpha. SNPs of PPP2R3C, a protein phosphatase, were associated with DI. SNP rs9322942 of *SRP54*, the gene for a signal recognition particle protein, is associated with adiponectin. Figure 2 shows the SNP association patterns with phenotypes RQ and TG at this QTL.

### The cis-SNP effects on gene expression

To test the function of the interrogated SNPs of the two adiponectin QTLs, we evaluated the effect of each variant on the levels of transcripts produced from the genes located within these two regions. We surveyed transcripts that were robustly detectable after filtering through our quality control procedures (see Methods). We then assessed the association between QTL-specific SNPs and each transcript annotated to the same locus.

**Figure 3:**
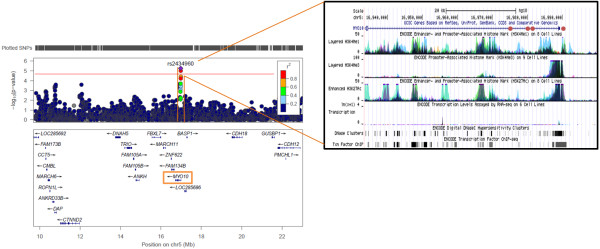
**Cis-effects of tagging SNPs located at chr5p14 on PWBC expression of *MYO10 *gene.** Association significance level (shown by –log_10_*p*-value) with *MYO10* gene expression of each tagging-SNP of the 1 LOD-score drop of adiponectin QTL at chr5: 9,792,000-23,021,100bp (NCBI36/hg18) is plotted against its respective genomic position using LocusZoom [35]. The position of each typed SNPs is also depicted in black bars above the plot. Red horizontal line shows the significance cutoff after Bonferoni-correction (p_α=0.05_=1.4×10^-5^). The color of the dot that represents each SNP on the plot shows the degree of correlation with rs2434960, the variant with the highest significance levels of cis-effect on *MYO10* expression. Correlation (r^2^) scale is depicted on the right. Genes mapped to this region are shown below the plot, with their directionality on the chromosome strand depicted. *MYO10* gene is framed in orange. In the blow-up figure on the right, functional genomics data from ENCODE consortium [33,34] of the region with the strongest cis-effect evidence in our analysis is shown in the UCSC Genome Browser view [31,32]. The four SNPs with the highest significance levels of cis-effects are labeled as red dots on *MYO10* gene track. ENCODE functional genomics tracks shown include evidence of enhancer- and promoter-associated histone modifications (H3K4Me1, H3K4Me3 and H3K27Ac), DNase hypersensitivity clusters and transcription factor binding assays.

Figure 3 shows the effects of SNPs of the chr5 locus on expression of *MYO10*. We found strong signals clustering in proximity to the transcription start site (TSS) of *MYO10*. SNPs exhibiting the strongest cis-effects (lowest p-value=5.43×10^-6^) are in high linkage disequilibrium (data not shown) and span a 17 kb region that has been shown previously to be highly important for transcription initiation [31-34].

We further analyzed the relationship between *MYO10* gene expression patterns in PWBC and key MetS phenotypes for which we had found evidence of SNP-phenotype associations (Table 2). Table 4 shows the levels of the correlations between the level of *MYO10* gene expression and MetS traits. We found positive correlations of *MYO10* expression with measures of adiposity. *MYO10* expression correlated negatively with plasma levels of adiponectin (although this did not reach statistical significance) and was positively correlated with plasma leptin at

**Table 4:** **Correlation (standardized beta ± SE) of PWBC expression levels of *MYO10 *transcript**

	**MetS phenotype**	***MYO10*(β±SE)**
**Body Composition**	Total Fat Mass (Fatkg), kg	0.04 ± 0.11
	Total Fat Mass (Fatpct), %	0.20 ± 0.11*
	Total Lean Mass (Leankg), kg	−0.06 ± 0.10
	Total Lean Mass (Leanpct), %	−0.20 ± 0.11*
**Adipokines**	Adiponectin, ng/mL	−0.02 ± 0.09
	Leptin, ng/mL	0.16 ± 0.09*

*Correlations with suggestive significance (p<0.1).

### Summary of candidate gene prioritization

Table 5 summarizes all the biologically relevant cancer or pro-inflammation genes, annotated by the SNP associations and gene expression correlations with MetS phenotypes under the adiponectin QTL peaks of 5p14 and 14q13. Figures 1 and 2 show SNP/phenotype association plots of SNPs located within these QTLs that we consider to be of the highest priority based on their biological relevance, in relation to the phenotypes from which the top candidate gene was identified.

**Table 5:** Summary of prioritized genes associating MetS with cancer or inflammation

**Biological mechanism**	**Annotated genes**	**Biological function**	**Association identifier phenotype**	**Relevance to cancer and/or inflammation**	**Evidence**
Cell cell adhesion and cell motility	*CDH18*	A member of Calcium-dependent cell adhesion molecules subfamily type 2 N-cadherin, affects morphogenesis, growth and tissue homeostasis.	weight, BMI, WC, HC and RQ	Formation, growth, invasion and migration of malignant tumors, including Crohn’s disease and prostate cancer. Ectopic expression in breast cancer cells promotes motility, invasion and metastasis.	SNP associations
	*CDH12*	A member of the cadherin family.	leptin	Its homologue, CDH11, promotes synovial fibroblasts to secret pro-inflammatory cytokines.	SNP associations
	*CTNND2*	A partner molecule in the cadherin complex. It is a transcription activator.	BMED	Involvement in cancer as above	SNP associations
	*MYO10*	An associate of the cadherin-catenin complex	Leankg, LDLppd, adiponectin, IL-6	Function unclear, presumably involved in cancer as above	SNP associations, SNP cis-effects on gene expression, expression/MetS phenotype correlations
Signal transduction	*AKAP6*	An anchor protein for the AMP-activated protein kinase, a regulator of glycogen, sugar and lipid metabolism.	HC, RQ and REE/Lean	It might be involved in any cancer biology that is mediated through p53	SNP associations
	*PRKD1*	A protein kinase that exerts its function by phosphorylating target proteins.	WHR, Fatpct, Leanpct, and TNF-alpha	It is involved in prostate cancer through E-cadherin phosphorylation	SNP associations
	*PPP2R3C*	It encodes the regulatory component of the protein ser/thr metallo-phosphatase. May regulate MCM3AP, which is a phosphorylation-dependent DNA replication initiation enzyme. Its homologue also play a role in the activation-induced cell death of B-cells.	DI	It may presumably be involved in cancer biology that relates MCM3. These include brain and thyroid cancer. It may also be involved in inflammation through its hypothetical function in B cell apoptosis.	SNP associations
Transcription	*TAG*	A novel tumor antigen gene that is immunogenic.	REE,FG, HOMA, HMED	Testis cancer, skin melanoma and myelogenous leukemia.	SNP associations
	*NPAS3*	A transcription factor which regulates neurogenesis, glucose metabolism and the linkage of the two.	Fatkg, Leankg, SubQF, TAF, REE/weight, TG, TC, cal. LDL-c, pulse, IL-1β, IL-6	It is a brain tumor suppressor and a marker for survival.	SNP associations,
					SNP cis-effects on gene expression
	*NKX2-8*	A homeobox transcription factor	Weight and height	Fetal liver and hepatocellular carcinoma and lung cancer	SNP associations
	*BRMS1L*	A homologue to transcription factor BRMS1, which functions in a histone acetylase complex that repress target genes	LMEDn and LDLppd	Breast cancer progression and melanoma metastasis	SNP associations
	*NOVA1*	It is a neuron-specific tumor antigen that has RNA-binding activity and functions to ensure correct pre-mRNA splicing of target RNA	BMI, WC, FG, FI, SI, AIR, HMED and sBP	It is involved in the paraneoplastic motor disorder	SNP associations
	*EGLN3*	It is a hydroxylase for target transcription factor HIF and mediates apoptosis in neuronal cells under development	IGR and LDL-c	It is involved in hemangioblastoma, clear cell renal carcinoma and formation of pheochromocytoma	SNP associations
Protein sorting	*MARCH11*	An ubiquitin ligase that is implicated in protein sorting and transport from trans-Golgi network to multivascular body.	Fatkg, FI, SI and IL-1β	Might be involved in pro-inflammatory cardiomyopathy.	SNP associations
	FBXL7	Another ubiquitin ligase specific for phosphorylated protein degradation.	WHR	Might be involved in pro-inflammation as above	SNP associations, SNP cis-effects on gene expression

## Discussion

As previously seen in other studies [36,37] plasma adiponectin was significantly correlated with several of the clinical outcome and biologic precursor phenotypes of MetS in our cohort. It correlated with BMI, total body fat mass, visceral vs. subcutaneous fat distribution, insulin to glucose response indices, levels of plasma lipids/lipoprotein density profiles, and circulating levels of leptin and cytokines (Table 1). As expected, the key clinical traits of MetS are also inter-correlated with each other in our subjects (Additional file 1: Table S1). Using adiponectin level as a surrogate for MetS, we previously identified two adiponectin-linked QTLs (5p14 and 14q13) in our study population. We now have used SNP association and the cis-effects of local SNPs on PWBC expression to discover MetS-related genes within these two QTLs. By doing so, we were able to identify multiple novel genes that have significant associations with individual MetS phenotypes. These genes can be mechanistically grouped into four categories (Table 5): (1) cell-cell adhesion and mobility (*CDH18*, *CDH12*, *CTNND2* and *MYO10*) (2) signal transduction (*AKAP*, *PRKD1* and *PPP2R3C*); (3) transcription (*NPAS3*, *BRMS1*, *Nova*-*1*, *EGLN3* and *TAG*); and (4) protein sorting (*MARCH11* and *FBXL7*). It is interesting that many of these genes are known to play a role in cancer biology. The link between MetS and increased cancer risk is well known [38] thus our prioritized genes provide a possible explanation for the observed association between MetS and cancer.

Our strongest SNPs associations on the chr5 QTL clustered around genes in the cell-cell adhesion pathways (Table 2 and Figure 1). Cell to cell adhesion is a key event in major cellular processes including proliferation, mobility, differentiation and cell death. It is also a basic mechanism facilitating the paracrine communication between cells. CDH18 is a cadherin that is expressed in multiple tissues, most prominently in the CNS [39] and is one of several cadherin genes located in this region of chromosome 5. The *CDH18* gene was most strongly associated with weight, BMI, waist circumference and RQ in our cohort, indicating a possible role in the development of body composition. Mechanistically this could be mediated via a role in CNS but since cell adhesion is a phenomenon that is integral to the regional expansion of adipose tissue it is possible that the gene may play a direct role in the preferential deposition of fat into the visceral abdominal region, a fundamental phenotype of MetS. Aberrations leading to alteration of cell adhesion are linked with several tumor types. N-cadherins specifically have been reported to play a role in the formation, growth, invasion [40] and migration of malignant tumors in various settings, including Crohn’s disease [41] and prostate cancer [42,43]. Deletions in the region of the chromosome 5 cadherin cluster have been specifically associated with risk of some malignancies [44]. Thus it is possible that the *CDH18* gene and other nearby genes identified in cohort (including *CTNND2*, *CDH12* and *MYO10*) play a mechanistic role in the observed connection between MetS and cancer risk.

Our strongest SNP association signals for plasma adiponectin levels clustered near the gene *MYO10* on chr5. *MYO10* protein is a member of the myosin family and has a role in trafficking adhesion molecules including integrins and adherins [45]. It is essential for the initiation, stability and formation of filopodia, a “finger-like” cellular protrusion that can sense environmental cues and a key structure for cell motility including cancer cells [46]. In a recent study of breast cancer with poor prognosis, expression of *MYO10* is significantly increased in patients with breast cancer. The expression of *MYO10* also differentially clustered with clinocopathological markers including p53 mutation, estrogen receptor (ER), tumor grade and patient survival [46]. In our analysis, variants in *MYO10* also associated with lean body mass, LDL diameter and IL-6, thus reflecting pleiotropy in its role in MetS, possibly via modulation of adiponectin levels. No data has shown that *MYO10* directly transports CDH18, however our data suggest that these two genes might work together in a biological pathway that predisposes individuals with MetS for carcinogenesis or high risk of metastasis. Our evaluation of the function of these tagging SNPs (or SNPs in LD with these) suggested strong cis-effects of variants near the TSS of *MYO10*. This region thus may harbor causal SNP(s) that directly affect binding of transcription factors. Results from human cell lines recently published by the ENCODE project has demonstrated that this 17 kb region is marked with transcription-activating histone modifications, is highly accessible to protein binding, and can be bound by multiple transcription factors in chromatin immunoprecipitation assays (Figure 3) [33,34]. Our approach therefore is able to efficiently identify genetic elements that can function in influencing genes important for our traits of interest.

At the QTL on chr14, SNP associations clustered near genes that function as signal transducers like *AKAP6* and transcription factors like *NPAS3* (Table 3 and Figure 2). The protein encoded by *AKAP6* is an anchor protein for the well-characterized protein kinase A (PKA), also known as AMP-activated protein kinase (AMPK). AKAPs compartmentalize AMPK, thereby regulating AMPK’s biological functions. AKAPs by themselves may also function as integrators of AMPK-related signal transduction pathways [47]. AMPK is a key regulator sensing cellular energy status and systemic energy balance [48] and mediates the effects of adipose tissue kinins including adiponectin and leptin in regulating body weight, glucose and lipid homeostasis [48]. It may also play a role in cancer via several processes including arresting of cell cycle progression, triggering inhibition of protein synthesis, and cell growth required in cell proliferation [49]. AMPK is also known to overcome growth factor signaling from a variety of stimuli mediated by the proto-oncogenes Akt and ERK. The AMPK signaling network also contains a number of tumor suppressor genes including p53, which is a universal tumor suppressor [50]. AMPK is thus one of the most prominent biological molecules proposed to connect metabolic pathways of MetS to cancer [50].

*NPAS3*, associated with several lipid and cytokine phenotypes in our cohort, encodes a neuronal basic helix-loop-helix (Per, Arnt, Sim) domain transcription factor, belonging to a family that has been reported to be crucial for neuronal development and maintenance [51] and exerts a regulatory role in neurogenesis and brain glucose metabolism [52]. Brain tissue of an *NPAS3* knockout mouse model shows altered levels of the glycolysis metabolites, the pentose phosphate shunt and Krebs cycle components, and aberrant behavior in mice. Glucose sensing by the brain is known to have effects on feeding, behavior, weight, weight control and associated metabolic consequences, as in MetS. *NPAS3* can also modulate cell cycle proliferation, apoptosis, migration and cell invasion and exhibits features of a tumor suppressor. Absence of *NPAS3* expression in astrocytomas is a negative prognostic marker for survival [53].

Increasing evidence has suggested that peripheral blood can be used to reveal the relative differences in gene expression among individuals [54,55]. Obtaining gene expression information using peripheral blood is simple and minimally invasive, making it possible to assay a large number of our subjects. Furthermore, obesity can be considered as a low-grade inflammation state [56], due to long-term low-level immune response triggered by accumulation of macrophages in visceral adipose tissue, chronic insulin resistance, adiponkine and cytokine production and malfunctional cardiovascular performance [57,58]. Profiling gene expression in peripheral blood therefore may capture changes in the expression of genes important for components of MetS such as visceral adiposity. In our analysis, we found some significant signals of SNP associations near pro-inflammatory genes. *MARCH11* (associated with fat, FI, SI and IL-1beta in our cohort) and *FBXL7* (associated with WHR in our cohort) belong to the family of ubiquitin ligases that are engaged in the process of protein sorting and transport of deformed ones from the trans-Golgi network [59,60]. *MARCH11* expression was shown in the Gene Expression Omnibus (GEO) database to be higher in patients with inflammatory dilated cardiomyopathy. *FBXL7*, an F-box protein family member, is specific for sorting of phosphorylated proteins and their degradation. Thus *MARCH11* and *FBXL7* may be involved in inflammation, a characteristic phenotype of MetS.

We do acknowledge that PWBC can reveal only a portion of the total gene expression information and that profiling it in other focal tissues like adipose and liver might reveal complementary genomic information. Further exploration of the expression levels of the other genes associated with MetS (and known to play a role in cancer biology) in other target tissues might help to reveal their gene expression correlations with MetS traits.

Our study has identified several novel as well as previously reported genes whose SNP variants are associated with phenotypes that are important components of MetS. e.g. genome-wide study of the genetic basis of insulin resistance reported that a variant of *AKAP6* was associated with BMI-adjusted fasting insulin (5×10^-7^) in a meta-analysis cohort of European descent [61]. *NPAS3* was found in the current study to be associated with a multitude of phenotypes including two inflammatory markers IL-1β and IL-6. Interestingly, in a recent report of a genome-wide search for associations between SNPs and biomarkers of systemic inflammation before and after an anti-inflammatory drug treatment, a variant of *NPAS3* was found to be one of the two most significantly associated gene loci with levels of C-reactive protein [62], and studies of visceral adiposity and BMI identified *FBXL7*, *MARCH6* and *PRKD1* as candidate loci [63-65].

## Conclusions

In the present study of a family cohort of Northern European origin, we conducted a comprehensive analysis using both SNP associations and gene expression correlations on two previously identified adiponectin QTLs and identified 14 novel genes associated with various metabolic syndrome traits. We found that many of these genes are involved in fundamental cellular functions that could provide a mechanistic link between the development of metabolic syndrome and cancer biology as well as systemic inflammation. Further studies are needed to elucidate the roles these genes play in the development of metabolic syndrome and its relationship with cancer biology. Eventually this could lead to the design of novel diagnostic markers and/or pharmaceutical agents for the prevention and treatment of MetS patients who are at higher risks for developing cancer and systemic inflammation.

## Competing interests

The authors declare that they have no competing interests.

## Authors’ contributions

Y.Z. analyzed data, contributed to discussion, wrote and revised the manuscript. J.W.K. performed statistical genetic analyses, contributed to discussion and co-wrote the manuscript. M.O. provided laboratory assistance in genotyping, contributed to discussion and data analysis. O.A. assisted in the phenotyping procedures, contributed to discussion and revision of the manuscript. D.C. assisted in data analysis and contributed to revision of the manuscript. R.M.A. assisted in the phenotyping procedures. U.B. facilitated the genotyping procedures. J.E.C. and M.A.C. did cytokine and transcriptome procedures. T.D.D. prepared the genotyping raw data for genetic analysis procedures. A.C. contributed to the original linkage analysis. H.H.H.G. assisted in the transcriptome data analysis. D.L.R. did the lipoprotein particle sizing. J.B. supervised and directed the overall statistical genetic analyses and contributed to discussion. A.H.K designed the study, analyzed data, contributed to discussion and revised the manuscript. All authors read and approved the final manuscript.

## Pre-publication history

The pre-publication history for this paper can be accessed here:

http://www.biomedcentral.com/1755-8794/6/14/prepub

## Supplementary Material

Additional file 1: Table S1 Intercorrelation analysis of leading MetS component phenotypes. Click here for file
